# Cancer Stem Cell Signaling during Repopulation in Head and Neck Cancer

**DOI:** 10.1155/2016/1894782

**Published:** 2016-01-06

**Authors:** George D. Wilson, Bryan J. Thibodeau, Laura E. Fortier, Barbara L. Pruetz, Sandra Galoforo, Brian Marples, Andrew M. Baschnagel, Jan Akervall, Jiayi Huang

**Affiliations:** ^1^Department of Radiation Oncology, William Beaumont Hospital, Royal Oak, MI 48703, USA; ^2^Beaumont BioBank, William Beaumont Hospital, Royal Oak, MI 48703, USA; ^3^Department of Human Oncology, University of Wisconsin Carbone Cancer Center, Madison, WI 53792, USA; ^4^Department of Otolaryngology, William Beaumont Hospital, Royal Oak, MI 48703, USA; ^5^Department of Radiation Oncology, Washington University School of Medicine in St. Louis, St. Louis, MO 63110, USA

## Abstract

The aim of the study was to investigate cancer stem signaling during the repopulation response of a head and neck squamous cell cancer (HNSCC) xenograft after radiation treatment. Xenografts were generated from low passage HNSCC cells and were treated with either sham radiation or 15 Gy in one fraction. At different time points, days 0, 3, and 10 for controls and days 4, 7, 12, and 21, after irradiation, 3 tumors per group were harvested for global gene expression, pathway analysis, and immunohistochemical evaluation. 316 genes were identified that were associated with a series of stem cell-related genes and were differentially expressed (*p* ≤ 0.01 and 1.5-fold) at a minimum of one time point in UT-SCC-14 xenografts after radiation. The largest network of genes that showed significant changes after irradiation was associated with* CD44*,* NOTCH1*, and* MET*. c-MET and ALDH1A3 staining correlated with the changes in gene expression. A clear pattern emerged that was consistent with the growth inhibition data in that genes associated with stem cell pathways were most active at day 7 and day 12 after irradiation. The* MET/CD44* axis seemed to be an important component of the repopulation response.

## 1. Introduction

Head and neck squamous cell cancers (HNSCCs) are a heterogeneous group of malignancies that originate in the mucosal lining of the upper aerodigestive tract. Despite advances in therapy, survival rates have remained static for many years [1].

The heterogeneity of HNSCC, as evidenced by histological, phenotypical, and karyotypical analyses [2, 3], has been mainly ascribed to the process of clonal expansion [4]. However, there is an increasing awareness that not all heterogeneity among cancer cells is the result of genetic variability and that, within a single tumor clone, cells have significantly different abilities to proliferate and form new tumors. This has led to the hypothesis that most cells in a cancer have a limited ability to divide and only a small subset of phenotypically distinct cells, the cancer stem cells (CSCs), have the capacity to self-renew and form new tumors [5]. The presence of cells with “stem-like” properties has been observed in HNSCC using a variety of different approaches [6–11].

For advanced inoperable HNSCC, treated with radiotherapy or chemoradiation, locoregional progression is the principal cause of treatment failure and cancer-related death. If tumor repopulation after therapy is a property of CSCs then the response of this population to radiation will be a critical constraint for curability. Several studies have provided evidence that CSCs are more resistant to radiation than their non-CSCs counterparts in a variety of cancers [12–15] and an association with chemoresistance has been reported in many studies including HNSCC [16].

In this study, we developed a model of local failure and repopulation in a HNSCC xenograft using a subcurative dose of radiation and studied the changes in protein expression of known stem cell-related genes as well as stem cell-related signaling pathways using global gene expression at key time points during the tumor response.

## 2. Materials and Methods

### 2.1. Cell Line, Xenografts, and Irradiation

The UT-SCC-14 cell line was obtained from Dr. R. Grénman, University of Turku, Finland, and was selected from a large panel of cell lines derived from primary and recurrent tumors from the head and neck region. The cell line has been maintained at low passage number such that it maintains phenotypic and morphological characteristics similar to the primary tumor which was a T3N1M0, moderately differentiated, HPV negative squamous cell carcinoma of the oral tongue. The experimental protocol was approved by the William Beaumont Hospital Animal Care Committee. Four- to 6-week-old female nude NIH III mice were used in these studies. Mice were caged in sterile housing in groups of five and were fed a diet of animal chow and water* ad libitum*. Xenografts were established by harvesting UT-SCC-14 cells in mid-log phase growth and injecting them subcutaneously into the flank of the mice, at a concentration of 2 × 10^6^ cells per 100 *μ*L of Matrigel (BD, Franklin Lakes, NJ). Tumor volume was measured by digital calipers and calculated using the standard formula (*πab*2)/6, where *a* is the largest and *b* is the smallest diameter. When the tumor volume reached a value of 300–400 mm^3^, animals were randomly assigned to the experiment groups. Tumors were measured three times each week. The endpoint of the experiment was when the tumors grew to a volume of 3,000 mm^3^. Animals were irradiated with a Faxitron Cabinet X-Ray System, Model 43855F (Faxitron X-Ray, Wheeling, IL), at a dose rate of 0.69 Gy/min, tube voltage of 160 KVp, and current of 4 mA. Animals were immobilized (without anesthetic) in a custom-designed jig that only exposed the hind flank to the radiation beam.

### 2.2. Experimental Design

Nine xenografts were randomized to receive sham treatment (control group) and twelve were randomized to receive 15 Gy (RT group). Groups of 3 mice from each treatment cohort were sacrificed at different time points after treatment. The control time points were selected during exponential growth of the unirradiated tumor and were not linked to the irradiated time points which were based on the regrowth characteristics of the tumors to yield observations during growth inhibition, transition to regrowth. At each time point the tumor was rapidly excised, bisected, and half snap-frozen and stored at −80°C whilst the other half was fixed in zinc formalin. For the control group, tumors were harvested at days 0, 3, and 10 after reaching the starting volume of 400–500 mm^3^. For the RT group, tumors were harvested at days 4, 7, 12, and 21 after treatment.

### 2.3. Isolation of RNA and Gene Expression

Laser capture microdissection was used to isolate cells from the peripheral regions of the tumor based on our previous observation of central necrosis after radiation treatment [17]. Frozen tissue samples were embedded in OCT (Tissue-Tek; Sakura Finetek, USA) and 8 *µ*m sections were cut and mounted onto PEN (polyethylene naphthalate) membrane glass slides (two sections per slide). Regions of periphery were identified on corresponding H&E slides of the tissue sections. The stained slides were microdissected within 2 hours of sectioning using an Arcturus^XT^ Microdissection System (Molecular Devices) onto CapSure Macro LCM Caps (Molecular Devices).

RNA isolation was carried out using RNeasy Plus Micro Kit (Qiagen, Valencia, CA). RNA concentration was determined on a ND-8000 spectrophotometer (NanoDrop Technologies, Inc., Wilmington, DE) and quality assessed on a Model 2100 Bioanalyzer (Agilent Technologies, Santa Clara, CA). High-quality RNA (i.e., RIN > 9.5) was used for the experiments. Differential mRNA expression analysis between cell populations was performed according to the GeneChip Whole Transcript (WT) Sense Target Labeling Assay Protocol (Affymetrix Inc., Santa Clara, CA) using 100 ng of RNA from each specimen. The fragments were hybridized overnight with the Human Exon 1.0 ST Array and then scanned with a GeneChip Scanner 3000. The GEO accession number is GSE61573.

### 2.4. Data Analysis

The  .CEL files containing the raw intensity data from the Affymetrix GeneChip arrays were imported into Partek Genomics Suite (version 6.6 beta, build 6.11.1115) and normalized using the robust multichip average with a guanine-cytosine content background correction, quantile normalization, log_2_-transformation, and median polish probeset summarization. Exons were then summarized to genes using the average of the probesets. Differentially expressed genes were identified using 1-way ANOVA comparing the samples from a given irradiated time point to the controls.

Gene set enrichment analysis (GSEA) and pathway analyses were performed using Pathway Studio 10.3 (Elsevier, New York, NY, USA). GSEA identifies highly regulated categories by considering all genes without any prefiltering based upon *p* value or fold change [18]. The expression microarray data was also analyzed using Pathway Studio's Sub-Network Enrichment Analysis (SNEA) tool [19, 20]. Pathway Studio utilizes MedScan, the literature mining program that searches publicly available literature such as PubMed for relationships between entities [21]. A subnetwork consists of a single seed (i.e., disease or cell process) and genes associated with this seed by regulation of/by the seed [22]. The expression microarray dataset is interrogated with no prior significance filtering, and enrichment of the subnetwork is determined by both the level of regulation in the network and the size of the network. The visualized subnetwork was limited to include only those genes that met *p* ≤ 0.01 and 1.5-fold change.

### 2.5. Immunohistochemistry and Image Analysis

4 *μ*m sections were cut, deparaffinized, and hydrated by passing through xylene followed by graded alcohol to distilled water. Antigen retrieval was achieved using antigen unmasking solution (pH 9.0) (Vector Labs, Burlingame, CA). Endogenous peroxide was blocked with Envision Flex peroxidase blocking solution (Dako, Carpinteria, CA) and the nonspecific staining blocked with CAS universal blocking solution (Life Technologies, Grand Island, NJ). Rabbit polyclonal antibodies for CD44 and ALDH1A3 were obtained from Abcam and used at a dilution of 1 : 50; c-MET clone 8F11 (Vector Laboratories Inc., Burlingame, California, USA) was diluted 1 : 100 and incubated at 42°C for 2 hours; (Abcam, Cambridge, MA) CD44 and ALDH1A3 were diluted 1 : 50 and 1 : 100, respectively, in antibody diluent containing PBS (pH 7.4), BSA, and 0.05% sodium azide and incubated for 60 minutes. After washing, the sections were incubated for 20 minutes using a dual rabbit/mouse polymer link detection kit (Dako) and visualized using diaminobenzidine and counterstained with hematoxylin. The sections were then dehydrated with a gradient of ethanol to xylene and coverslipped.

Images were captured at 10x magnification on a Nikon 90i microscope (Nikon, Melville, NY) equipped with a Nikon DS-Fi1 digital camera and Nikon Elements software. Ten images were captured from the peripheral areas of each tumor sample. The percent area of the image that stained positively for each protein was analyzed using ImageJ software (NIH). Images were split using the RGB function, an autothreshold was set on the blue image, and the area showing positive DAB staining was calculated using the measure function.

### 2.6. Statistical Analysis

Statistical analysis was carried out using Student's *t*-test. Data are presented as mean ± SE. A probability level of a *p* value of <0.05 was considered significant.

## 3. Results

### 3.1. Tumor Characteristics, Growth Rate, and Response to Radiation

**Figure 1:**
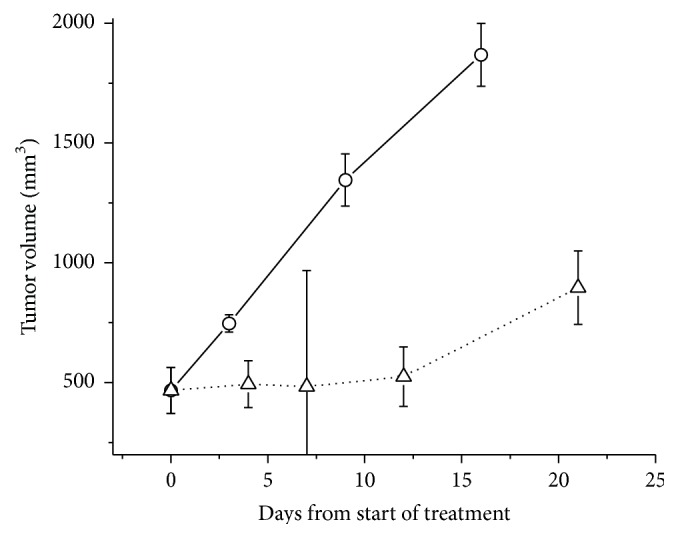
The tumor growth response of UT-SCC-14 xenografts to a dose of 15 Gy. Flank tumors were either irradiated with 15 Gy (∆) or sham-irradiated (O) and their growth was monitored over time with calipers.

Figure 1 shows the time course of the radiation response in the UT-SCC-14 xenografts. The untreated UT-SCC-14 xenografts had a volume doubling time of 4.8 ± 0.7 days whilst the irradiated tumors showed a period of profound growth arrest until day 12 after which they transitioned into repopulation. In a different aspect of this present study, we showed that early radiation necrosis (days 4–12) was characterized by central coagulative necrosis with pyknotic nuclei whilst late radiation necrosis (day 12 onwards) was characterized additionally by extensive necrosis with fragmentation and dystrophic calcifications [17]. The histological studies demonstrated that repopulation of the tumor occurred from the peripheral region, and this was the reason why immunohistochemical and gene expression analysis was restricted to only this region in the control and treated animals by the use of laser capture microdissection.

### 3.2. Immunohistochemical Staining of Stem Cell-Associated Proteins

**Figure 2:**
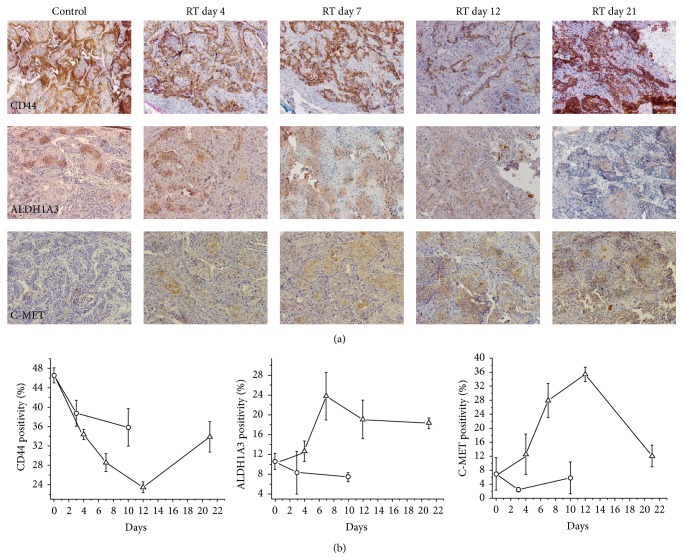
Immunohistochemical staining for CD44, ALDH1A3, and c-MET (a) and image analysis quantification of protein expression (b) after radiation (RT) treatment. CD44 was mainly associated with the cell surface and tended to stain cells in the outer layers of the tumor islands; its expression decreased after radiation but recovered between days 12 and 21. ALDH1A3 staining was cytoplasmic and showed a diffuse pattern throughout the tumors. After irradiation ALDH1A3 increased to a maximum on day 7 and remained elevated for the period of observation. In some areas cells became more intensely stained with ALDH1A3 after irradiation. c-MET showed a staining pattern which was similar to ALDH1A3 being diffuse and cytoplasmic. The levels of c-MET increased at day 4 and reached a maximum at day 12 after which they declined rapidly to control levels (×20).

Figure 2(a) shows the immunohistochemical staining pattern and changes after irradiation of 3 of the genes with known association to stem cells in HNSCC; in Figure 2(b), the quantitative analysis of these genes is presented. CD44 was mainly associated with the cell surface and tended to stain cells in the outer layers of the tumor islands formed by the UT-SCC-14 cells; no staining was observed in the stroma. Following radiation, there was not a great change in the intensity of staining but the number of positive cells fell steadily at day 4 (*p* = 0.0201) and day 7 (*p* = 0.0123) and to a nadir at day 12 (*p* = 0.0014) after which expression levels recovered. The staining pattern became more predominantly associated with the outermost cell layers of the tumor islands. In control tumors there was a reduction in CD44 expression as tumor volume increased. Interestingly, CD44 expression decreased in control tumors as a function of time. Histologically, this was associated with a tendency for the larger tumors to show differentiation and increased keratinization.

ALDH1A3 staining was cytoplasmic and showed a diffuse pattern throughout the tumors where complete tumor islands were often positive with little evidence of any preferential peripheral staining within those islands. After irradiation ALDH1A3 increased to a maximum on day 7 (*p* = 0.047) and remained elevated for the period of observation. In some areas cells became more intensely stained with ALDH1A3 after irradiation.

Intrinsic levels of c-MET were relatively low in UT-SCC-14 xenografts and showed a staining pattern which was similar to ALD1A3 being diffuse and cytoplasmic. The levels of c-MET increased at day 7 (*p* = 0.0357) and reached a maximum at day 12 (*p* = 0.005) after which they declined rapidly to control levels.

### 3.3. Global Gene Expression Changes Associated with Stem Cell-Related Signaling

In Table 1, Pathway Studio software was used to identify genes associated with a series of stem cell-related genes* CD44*,* ALDH*,* MET*,* NOTCH1*,* BMI1*,* OCT3/4*,* NANOG*,* SOX2*, and* CD133*, which were differentially expressed (*p* ≤ 0.01 and 1.5-fold) at a minimum of one time point in UT-SCC-14 xenografts after radiation. The associated genes could be of any category including ligand, transcription factor, positive or negative regulator, or receptor. Pathway Studio identified 316 genes that met these criteria for the 9 stem cell-related genes, many of which were shared between the target genes. The largest network of genes that showed significant changes after irradiation was associated with* CD44*,* NOTCH1*, and* MET*. The smallest network of genes was associated with* ALDH*. There was a clear pattern that emerged in which genes associated with stem cell pathways were most active at day 7 and day 12 after irradiation. At day 7, the tumors are still in a state of profound growth inhibition whilst at day 12 the transition into regrowth is becoming apparent (Figure 1). At days 4, 7, and 12, there was a higher proportion of genes that were upregulated in the stem cell-associated pathways but, interestingly, at day 21 (during active regrowth) more downregulated genes were represented.

### 3.4. Specific Gene Expression Changes Associated with Stem Cell Signaling

Three genes,* CTNNB1*,* MMP9*, and* NOTCH1*, were identified in the pathways of all of the nine stem cell-associated genes. However,* CTNNB1* was only upregulated at day 4;* MMP9* was upregulated at days 12 and 21, whilst* NOTCH1* was downregulated at days 12 and 21. Three genes,* IL6*,* IL8*, and* SMAD2*, were represented in 8 of the 9 signaling pathways.* SMAD2* was only upregulated at day 7 whilst both* IL6* and* IL8* were upregulated at day 12. Seven genes were present in 7 of the 9 pathways and included* ICAM1*,* LIF*,* MAPK8*,* MET*,* SPP1*,* TGFA*, and* TGFBR2*.* MET* and* TGFA* were upregulated at key days 7 and 12.* LIF1* and* SPP1* were upregulated at day 12 whilst* MAPK8* was upregulated at day 7 only.

### 3.5. Specific Gene Expression Changes Associated with* CD44*

Supplemental Table 1 in Supplementary Material available online at http://dx.doi.org/10.1155/2016/1894782 shows genes significantly altered at one time point or more known to be associated with* CD44* signaling. It is clear that the greatest activity centers on days 7 and 12. On day 7 the genes showing the greatest fold change were as follows:* TIMP3* (+2.44),* TIMP2* (+2.39),* IGHMBP2* (+2.36),* MMP3*,* KHDRB53*,* TGFA*, and* WIPF1* all showed between 1.90- and 1.99-fold increases whilst* PTGES*,* PLAUR*,* CTSB*,* MET*, and* VANGL1* all showed greater than 1.75-fold increases. The top downregulated genes were* IGFBP5* (−3.40),* PTTG1* (−3.07),* EGR1* (−2.55), and* FASN* (−2.54). On day 12 more genes showed significant alterations and higher levels of fold change.* IL8* (+6.00),* HAS2* (+5.35),* ICAM1* (+3.51),* PLAUR* (+3.32),* LIF* (+3.13), and* LYN* (+3.07) were the most significantly upregulated genes but* TIMP2*,* MMP9*,* HBEGF*,* CXCL16*,* SLC7A11*,* MME*,* EPCAM*,* CTSS*,* PLAU*, and* TGFBR2* all showed greater than 2-fold upregulation. Of the downregulated genes* IGFBP5*,* BCAM*, and* NOTCH1* showed the greatest downregulation. Interestingly* HRAS* was downregulated on days 7, 12, and 21.

### 3.6. Specific Gene Expression Changes Associated with* MET*

Supplemental Table 2 shows that* MET* shares many common genes with* CD44* and shows a similar pattern of the most active gene expression changes taking place on days 7 and 12. Some of the key genes upregulated on day 7 were* WNT7A* (+2.46),* TIMP3* (+2.44),* TIMP2* (+2.39),* MMP3* (+1.99),* TGFA* (+1.91), and* PLAUR* (+1.84).* MET* itself was upregulated 1.76-fold and was also upregulated at day 12 1.86-fold. Several genes were highly upregulated in the c-MET pathway at day 12 including* LCN2* (+10.0),* IL8* (+6.0),* ICAM1* (+3.51),* PLAUR* (+3.32),* LIF* (+3.13),* LYN* (+3.07),* TIMP2* (+2.92),* MMP9* (+2.82),* HBEGF* (+2.76), and* WNT7A* (+2.25). Some of the most noticeable downregulated genes were* HRAS*,* FASN*,* EGR1*,* FGFR3*, and* NOTCH1*.

### 3.7. Specific Gene Expression Changes Associated with* NOTCH1*

Supplemental Table 3 shows that* NOTCH1* also shares many common genes with* CD44* and* MET* and reflects the changes in* WNT7A*,* TIMP3*,* TIMP2*,* MMP3*,* TGFA*,* PLAUR*, and* MET* at day 7. Other genes upregulated in this pathway at day 7 included* FHL1* (+2.45),* NFATC2* (+2.04), and* ENO2 *(+2.03). Similarly, at day 12 many mutual genes were upregulated including* IL8*,* ICAM1*,* PLAUR*,* LIF*,* TIMP2*,* MMP9*,* HBEGF*, and* WNT7A*; other notable upregulated genes included* IL1RL1*,* FHL1*,* IL6ST*,* TRIB3*,* PMAIP1*,* RIPK2*,* PLAU*,* TGFBR2*,* TGFA*,* BIRC3*, and* BMI1*.* IGFBP5*,* NOTCH1*,* JAG2*,* HRAS*, and* STAT6* were notable genes downregulated at day 12.

### 3.8. Specific Gene Expression Changes Associated with Other Stem Cell-Related Genes including* ALDH*,* BMI1*,* CD133*,* NANOG*,* POU5F1*, and* SOX2*

The* ALDH*,* BMI1*,* CD133*,* NANOG*,* POU5F1*, and* SOX2* pathways had fewer members which were significantly altered after radiation (Supplemental Tables 4–9).* ALDH1A3* was the key gene in the* ALDH* pathway that was upregulated at day 7 (+2.18), day 12 (+4.18), and day 21 (+2.75). The* BMI1* pathway showed nothing remarkable outside of genes altered in many of the other pathways. There was a similar conclusion with the* CD133* pathway. The* NANOG* and* POU5F1 (OCT 3/4)* pathways shared some similar upregulated genes which were not associated with the other major signaling pathways. These included* TOR1AIP2* which was upregulated at day 4 (+3.5), day 7 (+4.27), and day 12 (+3.75) and* PMP22* which was upregulated at day 7 (+3.32), day 12 (+3.02), and day 21 (+1.95).* SOX2* signaling was relatively unremarkable with most genes being represented in the other major pathways.

### 3.9. Common Cell Processes Associated with Stem Cell Signaling after Radiation Treatment

**Figure 3:**
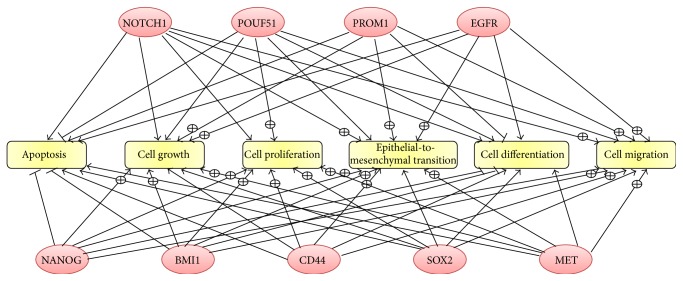
Cellular processes common to all nine cancer stem cell- (CSC-) associated genes. Pathway Studio was used to identify known relationships and cellular processes that are common between the CSC-associated genes. The ⨁ symbol on the arrow represents positive regulation of the process whilst a – through the arrow represents negative regulation.

Figure 3 shows cell processes that were linked with all nine stem cell-associated genes. Apoptosis and differentiation were almost universally negatively regulated by the genes whilst cell growth, cell proliferation, cell migration, and epithelial-to-mesenchymal transition were positively regulated by the stem cell-associated genes.

### 3.10. Epithelial-to-Mesenchymal Transition

**Figure 4:**
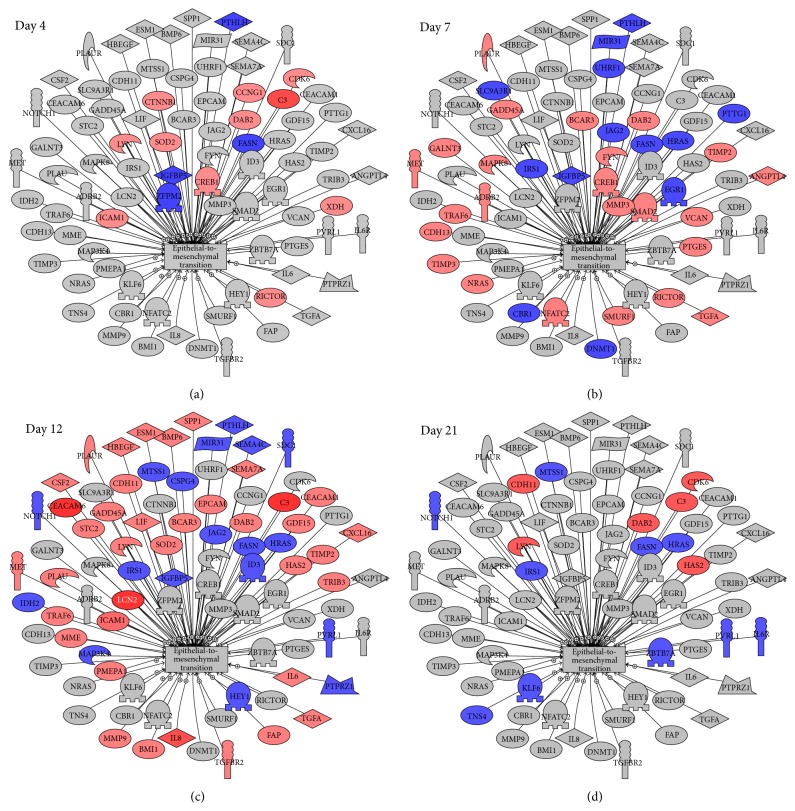
Changes in genes associated with epithelial-to-mesenchymal transition after radiation. Using Pathway Studio, genes known to be associated with epithelial-to-mesenchymal transition and differentially expressed at a minimum of one time point after radiation with a *p* value ≤ 0.01 and fold change (negative or positive) of 1.5 or greater were identified. Red represents genes upregulated after irradiation whilst blue represents downregulated genes.

Epithelial-to-mesenchymal transition (EMT) appears as a common cell process in Figure 3, and it functions to diversify cell types during embryogenesis and also allows epithelial cells to acquire a migratory, mesenchymal-like phenotype during wound healing. Inducing EMT in HNSCC cells correlates with the emergence of CSCs and vice versa. In Supplemental Table 10, we have listed the genes showing *p* ≤ 0.01 and at least a 1.5-fold change after irradiation. There is a similar pattern to the stem cell-associated genes where significant changes increase from day 4 through day 7, peak at day 12, and decline by day 21. Many of the genes are shared among the stem cell-associated gene pathways. In Figure 4, we have linked the pathways of the stem cell-associated genes with EMT presenting common genes found in at least 5 of the 9 CSC markers and EMT with a differential expression of *p* ≤ 0.01 and at least a 1.5-fold change.* CTNNB1* and* MMP9* were the only genes linked to all the subnetworks whilst* IL8*,* IL6*, and* SMAD2* were found in 9 of the ten subnetworks and* ICAM1*,* SPP1*,* TGFA*,* LIF*,* TGFBR2*, and* MAPK3* were found in 8 subnetworks.

## 4. Discussion

It is generally accepted that CSC populations with self-renewal and differentiation capacities exist within HNSCC [16, 23]. A better understanding of the mechanisms that govern the dynamics of HNSCC CSCs is needed to unravel their importance and role in treatment resistance and disease progression. In this study, we developed a model which interrogated the molecular changes associated with known stem cell linked genes during the repopulation/regrowth of a HNSCC xenograft after a subcurative radiation treatment. The underlying rationale for this approach was that if the CSCs were important in treatment resistance, they would dominate the repopulation response as a single dose of 15 Gy would kill approximately 90% of the cells in the tumor.

Many of the genes including CD44 [24], BMI1 [25], c-MET [26], NOTCH1 [27], ALDH1 [28], and SOX2 [29] have been associated with poor prognosis in HNSCC. However, the genes were specifically chosen in this study because they have all been associated with CSCs in HNSCC and other cancers. CD44 is a multifunctional transmembrane glycoprotein that is a receptor hyaluronic acid but can also interact with several additional molecules such as fibronectin, fibrinogen, laminin, galectin-8, collagen, chondroitin sulfate, and osteopontin. It has been proposed that HA binding to CD44 promotes its association with EGFR as well as EGFR phosphorylation. CD44 is the most commonly used cell-surface marker for CSCs across multiple tumor types and has been consistently acknowledged as CSC marker in HNSCC [30, 31]. Prince and colleagues were the first to show CD44^+^ cells were highly tumorigenic and successfully propagated in serial transplantation studies [10]. CD44 expression has been associated with poor prognosis in HNSCC [24]. The aldehyde dehydrogenase family of enzymes participates in retinoic acid biosynthesis and is an integral component of squamous epithelia differentiation. Cells which express high levels of ALDH1, extracted from primary HNSCC, were shown to be more tumorigenic in establishing xenografts than cells with low expression [6] and other studies have shown that ALDH1 overexpression correlates with poor prognosis [32]. The combination of CD44^+^ and ALDH^high^ expression further identified primary HNSCC cells that showed enhanced xenotransplantation efficiency [33]. c-MET is a receptor tyrosine kinase that has been implicated in the progression of HNSCC [34] and associated with worse response to treatment and prognosis [26]. It has also been implicated in CSCs as a subset of c-MET^+^ cells were shown to have enhanced tumorigenicity which was more pronounced when cells which were positive for both c-MET and CD44 were transplanted [35]. NOTCH1 signaling ordinarily drives keratinocyte differentiation in squamous epithelia [36] but takes on stemness-promoting activity upon malignant transformation [37]. The expression of NOTCH1 and *β*-catenin has been reported to be increased in CD44^+^ HNSCC cells [38]. CD133 (also known as AC133 or prominin-1) is a cell-surface glycoprotein comprising five transmembrane domains and two large glycosylated extracellular loops and has commonly been associated with subpopulations of cells with highly tumorigenic capacity in several cancers including HNSCC [39]. BMI1 is a member of the Polycomb family of transcription repressors. Emerging studies show that BMI1 has an important function as a biomarker of CSCs and is associated with self-renewal characteristics, tumor initiation, progression, invasion, metastasis, tumor recurrence, and resistance to chemotherapy and radiotherapy [40]. NANOG, POU5F1 (Oct-3/Oct-4), and SOX2 are transcription factors that are important in the maintenance of pluripotency and self-renewal in embryonic stem cells [41]. All these genes have been implicated in cancer stem cell traits in HNSCC [42].

There was a clear sequence of events that occurred in the signaling pathways for each of the stem cell-associated genes in that the number of significantly altered components increased from day 4 after irradiation to day 7, peaked at day 12, and returned to near control levels by day 21. This sequence could be interpreted to represent the initial killing of radiation-sensitive cells, the recovery dominated by changes in the surviving radiation-resistant stem cell population, and then the return to normal activity driven by differentiation of stem cells into the original tumor phenotype. However, this is speculation as the changes in subpopulations of cells within the tumor cannot be verified by the current experimental design. The only principal gene that was upregulated at days 7 and 12 was* MET* (1.76- and 1.86-fold, resp.), whilst the* ALDH1* family member* ALDH1A3* was upregulated at day 7 (+2.18), day 12 (+4.18), and day 21 (+2.75). Both these genes also showed increased protein expression as evidenced by immunohistochemistry (Figure 2).* BMI1* was modestly upregulated at day 12 (+1.53) whilst* NOTCH1* was downregulated at day 12 (−2.11). The likely sequence of events following a single dose of 15 Gy would be initial killing of radiosensitive cells followed by a prolonged cell cycle delay with further rounds of cell death as cells attempt mitosis in the presence of unrepaired DNA damage. Accelerated repopulation during radiation has been described for many years [43] but the underlying stimulus for repopulation has not been well studied at the mechanistic level [44, 45]. This is the first study to specifically look at stem cell signaling during the process of repopulation in an* in vivo* tumor model.

Each stem cell-associated gene was characterized by its own network of ligands, transcription factors, positive or negative regulators, or receptors which were significantly altered on different days after radiation treatment. However, there were genes that were common to many of the stem cell-associated genes including* CTNNB1*,* MMP9*,* IL8*,* IL6*,* SMAD2*,* ICAM1*,* SPP1*,* TGFA*,* LIF*,* TGFBR2*, and* MAPK3*. The key time period is likely to be the events and signaling that takes place between day 7 and day 12. At this time, the tumor (and stromal) cells will be recovering from the single large radiation dose and the pathways that are activating the repopulation response will become dominant. This time period witnessed the most activity in terms of significantly altered genes linked with stem cell-associated genes as well as with the epithelial-to-mesenchymal transition. Interestingly, the NOTCH pathway did not seem to be activated following radiation; the gene* NOTCH1* was downregulated at day 12, one of its canonical transmembrane ligands* JAG2* was downregulated at days 7 and 12, and its target gene* HEY1* was also downregulated at day 12. The NOTCH pathway is one of the most intensively studied candidate genes involved in CSCs, and NOTCH signaling has been reported to promote the self-renewal of CSC in several malignancies and to participate in tumor-stroma and tumor-endothelium interactions in CSC niches in primary and metastatic tumors [46]. Another gene which has been reported to be important in CSCs in head and neck cancer [40],* BMI1*, also did not show any significant changes during the repopulation response.

One of the most intriguing findings was centered on* ALDH1*. This gene has been consistently associated with CSCs in head and neck cancer [47, 48]. In our study* ALDH1A3* was significantly upregulated at days 7, 12, and 21 as were the protein levels by immunohistochemistry (Figure 2), yet there were very few other family members that showed significant changes (Supplemental Table 4). ALDH is an intracellular enzyme that is normally active in the liver. Its primary functions are retinol conversion to retinoic acid and the oxidation of toxic aldehyde metabolites, such as those formed during the alcohol metabolism and certain chemotherapeutic drugs. It would seem to be a very significant biomarker for the identification of stem cells but offers little opportunities to design drugs to target its associated pathways. However, adoptive therapy with ALDH1A1-specific CD8(+) T cells is a promising approach to target CSCs based on this biomarker [49].

The* MET/CD44* axis seems to be an important component of the CSC response in this HNSCC tumor model. Like* ALDH1*,* MET* gene expression and protein expression (Figure 2) were significantly altered at the key time points after irradiation. However,* CD44* gene expression was not changed, and CD44 protein initially decreased following radiation before showing a recovery at day 12. c-MET signaling plays a critical role in tumor progression, invasion, and metastasis, and we have recently shown it to be associated with poor prognosis in locally advanced HNSCC patients treated with chemoradiation [26]. Sun and Wang showed that c-MET(+) HNSCC cells increased expression of self-renewal pathways, were spared by cisplatin treatment, and were responsible for mediating metastasis [35]. When CD44 expression was taken into account in addition to c-MET, the dual biomarker expressing population showed enhanced tumorigenicity [35]. A recent study showed that hepatocyte growth factor (the only known ligand for c-MET) stimulation increased the self-renewal and expression of stemness markers, such as Oct4, Sox2, Nanog, and CD44, in HNSCC stem-like cells [50]. In addition, knockdown of c-MET attenuated CSC traits, augmented cisplatin chemosensitivity, and inhibited xenotransplantation efficiency. Inhibiting c-MET using a kinase inhibitor, SU11274, inhibited sphere formation and suppressed the transcriptional levels of Oct4 and Sox2 of HNSCC stem-like cells [50]. It is difficult to decipher which are the key genes involved in the* MET/CD44* axis, but IL-8 has been shown to act as an autocrine growth factor in HNSCC, melanoma, and lung carcinoma and addition of recombinant IL-8 can promote directly the proliferation of HNSCC cell lines [51].

EMT is also linked with the acquisition of stem cell-like characteristics. The concept of EMT inducing a CSC phenotype provides a possible mechanistic basis for metastasis, chemoresistance, tumor dormancy, and delayed recurrence. It is difficult to recognize the morphological cellular changes associated with EMT after radiation due to the cellular changes induced by the treatment which include enlarged nuclei with degenerative atypia, smudged chromatin, pyknotic nuclei, and micro-/macrovesicular vacuolation of cytoplasm [17]. However, the gene expression analysis highlighted the importance of EMT-associated genes in the repopulation response suggesting that this cellular process is a key component of the tumor response to recovery from cytotoxic damage.

In summary, stem cell-associated gene signaling is a key component of the response of HNSCC to DNA damage and may be driver of the repopulation response of the tumor. The limitation of this study is that experiments were performed on one tumor model and further work will be required using different HNSCC tumor models to validate these findings. In addition, the model used a single dose of irradiation; further work will be required using fractionation schedules with different total doses to confirm these findings in a clinically realistic schedule. With these limitations, this study indicates that the* MET/CD44* axis would seem to be an important contributor to the recovery/regrowth response.

## 5. Conclusion

Although CSCs have been consistently identified in HNSCC and shown to be capable of enhancing tumorigenicity and resistance to chemotherapy, there has been no study that attempts to decipher their importance in the repopulation/regrowth response of HNSCC to radiation treatment. In this study, we have demonstrated that several pathways associated with known stem cell-associated genes show a similar response after irradiation that is consistent with the transition from damage recovery to regrowth of a HNSCC xenograft* in vivo*. The study highlights the potential importance of the* MET/CD44* axis as well as the importance of epithelial-to-mesenchymal regulators in the recovery process after DNA damage caused by radiation.

## Supplementary Material

The supplementary material consists of 10 tables which list the differentially expressed genes (p ≤ 0.01 and 1.5-fold) at a minimum of one time point after radiation for CD44, c-MET, NOTCH, ALDH1, BMI1, CD133, NANOG, POU5F1, SOX2 and epithelial to mesenchymal transition.

## Figures and Tables

**Table 1:** Genes differentially expressed (*p* ≤ 0.01 and 1.5-fold) at a minimum of one time point in UT-SCC-14 xenografts after radiation.

Gene	*n*	Day 4		Day 7		Day 12		Day 21	
		↑	↓	↑	↓	↑	↓	↑	↑
CD44	62	5	3	20	12	28	11	3	4
ALDH	9	2	0	2	4	3	1	1	1
MET	48	7	1	16	8	25	9	4	5
OCT3/4	37	6	1	10	5	18	4	4	4
BMI1	16	1	0	2	7	6	1	0	1
NOTCH1	66	7	4	19	14	29	11	1	4
NANOG	29	5	2	7	4	15	4	4	3
SOX2	29	3	1	8	5	12	3	1	2
CD133	20	2	1	3	4	9	3	0	3
Total	**316**	**38**	**13**	**87**	**63**	**145**	**47**	**18**	**27**

Using Partek Genomics software and Pathway Studio, genes that were differentially expressed at a minimum of one time point after radiation with a *p* value ≤ 0.01 and fold change (negative or positive) of 1.5 or greater were identified for each of the CSC-associated genes. For each gene, *n* represents the total number of identified genes in each pathway whilst the ↑ and ↓ arrows for each day after radiation represent the number of genes which were upregulated or downregulated, respectively.
