# Supplementary material for: Cancer Stem Cell Signaling during Repopulation in Head and Neck Cancer
Source: Stem Cells Int. 2016 Jan 6;2016:1894782. doi: 10.1155/2016/1894782 (PMC4736761; doi:10.1155/2016/1894782)
Supplement: Supplementary file 1 — The supplementary material consists of 10 tables which list the differentially expressed genes (p ≤ 0.01 and 1.5-fold) at a minimum of one time point after radiation for CD44, c-MET, NOTCH, ALDH1, BMI1, CD133, NANOG, POU5F1, SOX2 and epithelial to mesenchymal transition. [file 1894782.f1.pdf]

Supplemental Table 1. CD44 signaling. Differentially expressed (p≤0.01 and 1.5-fold) at a minimum of one time point in UT14 xenografts after radiation. Bold is upregulated after XRT; red, downregulated.

Day 4 vs Controls		Day 7 vs Controls		Day 12 vs Controls		Day 21 vs Controls	
**ICAM1**	**2.58**	**TIMP3**	**2.44**	**IL8**	**6.00**	**HAS2**	**4.82**
**LYN**	**2.02**	**TIMP2**	**2.39**	**HAS2**	**5.35**	**LYN**	**2.19**
**SOD2**	**1.79**	**IGHMBP2**	**2.36**	**ICAM1**	**3.51**	**KHDRBS3**	**1.78**
**CTNNB1**	**1.63**	**MMP3**	**1.99**	**PLAUR**	**3.32**	LAMA5	-1.55
**CREB1**	**1.55**	**KHDRBS3**	**1.98**	**LIF**	**3.13**	NOTCH1	-1.69
BCAM	-1.77	**TGFA**	**1.91**	**LYN**	**3.07**	HRAS	-1.71
IGFBP5	-1.85	**WIPF1**	**1.90**	**TIMP2**	**2.92**	FASN	-1.74
FASN	-2.06	**PTGES**	**1.86**	**MMP9**	**2.82**		
		**PLAUR**	**1.84**	**HBEGF**	**2.76**		
		**CTSB**	**1.83**	**CXCL16**	**2.64**		
		**MET**	**1.76**	**SLC7A11**	**2.38**		
		**VANGL1**	**1.76**	**MME**	**2.17**		
		**CD59**	**1.73**	**EPCAM**	**2.10**		
		**FYN**	**1.72**	**CTSS**	**2.09**		
		**ANGPTL4**	**1.69**	**PLAU**	**2.02**		
		**VCAN**	**1.69**	**TGFBR2**	**1.97**		
		**SMAD2**	**1.56**	**FAP**	**1.91**		
		**MAPK8**	**1.55**	**SOD2**	**1.87**		
		**CREB1**	**1.53**	**MET**	**1.86**		
		**ADRB2**	**1.53**	**CEACAM1**	**1.85**		
		BDKRB2	-1.52	**TLR2**	**1.85**		
		PRKCA	-1.53	**CSF1**	**1.83**		
		C1QBP	-1.60	**TGFA**	**1.73**		

	HRAS	-1.61	**BIRC3**	**1.62**	
	SLC9A3R1	-1.79	**IL6**	**1.61**	
	LAMA5	-1.90	**CSF2**	**1.57**	
	DNMT1	-1.93	**SPP1**	**1.56**	
	TFRC	-2.02	**CD59**	**1.56**	
	FASN	-2.54	BDKRB2	-1.58	
	EGR1	-2.55	SDC1	-1.70	
	PTTG1	-3.08	HRAS	-1.77	
	IGFBP5	-3.40	LAMA5	-1.78	
			LRP1	-1.80	
			FASN	-1.88	
			LDB1	-2.00	
			CSPG4	-2.09	
			NOTCH1	-2.11	
			BCAM	-2.29	
			IGFBP5	-2.55	

Supplemental Table 2. c-Met signaling. Differentially expressed (p≤0.01 and 1.5-fold) at a minimum of one time point in UT14 xenografts after radiation. Bold is upregulated after XRT; red, downregulated.

**Day 4 vs Controls**		**Day 7 vs Controls**		**Day 12 vs Controls**		**Day 21 vs Controls**	
**ICAM1**	**2.58**	**WNT7A**	**2.46**	**LCN2**	**10.0**	**LYN**	**2.19**
**LYN**	**2.02**	**TIMP3**	**2.44**	**IL8**	**6.00**	**SNX2**	**1.83**
**PIK3R1**	**1.76**	**TIMP2**	**2.39**	**ICAM1**	**3.51**	**PIK3R1**	**1.59**
**CTNNB1**	**1.63**	**MMP3**	**1.99**	**PLAUR**	**3.32**	**CDK6**	**1.54**
**CDK6**	**1.61**	**TGFA**	**1.91**	**LIF**	**3.13**	IRS1	-1.66
**RICTOR**	**1.56**	**PLAUR**	**1.84**	**LYN**	**3.07**	NOTCH1	-1.69
**CREB1**	**1.55**	**CDH13**	**1.80**	**TIMP2**	**2.92**	HRAS	-1.71
FASN	-2.06	**MET**	**1.76**	**MMP9**	**2.82**	FASN	-1.74
		**FYN**	**1.72**	**HBEGF**	**2.76**	FGFR3	-2.20
		**VCAN**	**1.69**	**WNT7A**	**2.25**		
		**ATF6**	**1.67**	**CTSS**	**2.09**		
		**RICTOR**	**1.59**	**PLAU**	**2.02**		
		**SMAD2**	**1.56**	**TGFBR2**	**1.97**		
		**MAPK8**	**1.55**	**SH3KBP1**	**1.91**		
		**CREB1**	**1.53**	**C1GALT1**	**1.89**		
		**SH3KBP1**	**1.50**	**MET**	**1.86**		
		PRKCA	-1.53	**IRF1**	**1.84**		
		MIR31	-1.55	**SNX2**	**1.82**		
		HRAS	-1.61	**TGFA**	**1.73**		
		IRS1	-1.64	**ATF6**	**1.73**		
		PLXNB1	-1.85	**ESM1**	**1.72**		
		CASP1	-1.87	**PIK3R1**	**1.61**		
		FASN	-2.54	**IL6**	**1.61**		

	EGR1	-2.55	**CSF2**	**1.57**	
			**SPP1**	**1.56**	
			MIR31	-1.56	
			SDC1	-1.70	
			HRAS	-1.77	
			IRS1	-1.84	
			FASN	-1.88	
			PLXNB1	-1.99	
			CSPG4	-2.09	
			NOTCH1	-2.11	
			FGFR3	-2.72	

Supplemental Table 3. Notch signaling. Differentially expressed (p≤0.01 and 1.5-fold) at a minimum of one time point in UT14 xenografts after radiation. Bold is upregulated after XRT; red, downregulated.

**Day 4 vs Controls**		**Day 7 vs Controls**		**Day 12 vs Controls**		**Day 21 vs Controls**	
**ICAM1**	**2.58**	**WNT7A**	**2.46**	**IL8**	**6.00**	**CDK6**	**1.54**
**APEX1**	**1.75**	**FHL1**	**2.45**	**ICAM1**	**3.51**	EFNB1	-1.52
**IRAK2**	**1.72**	**TIMP3**	**2.44**	**PLAUR**	**3.32**	ZBTB7A	-1.66
**CTNNB1**	**1.63**	**TIMP2**	**2.39**	**LIF**	**3.13**	NOTCH1	-1.69
**CDK6**	**1.61**	**FTL**	**2.21**	**TIMP2**	**2.92**	HRAS	-1.71
**RICTOR**	**1.56**	**NFATC2**	**2.04**	**MMP9**	**2.82**		
**CREB1**	**1.55**	**ENO2**	**2.03**	**HBEGF**	**2.76**		
DACH1	-1.59	**MMP3**	**1.99**	**IL1RL1**	**2.50**		
LRP8	-1.65	**TGFA**	**1.91**	**WNT7A**	**2.25**		
IGFBP5	-1.85	**PLAUR**	**1.84**	**FHL1**	**2.22**		
PTHLH	-2.17	**DLG1**	**1.81**	**IL6ST**	**2.11**		
		**TP53BP2**	**1.81**	**TRIB3**	**2.10**		
		**MET**	**1.76**	**PMAIP1**	**2.04**		
		**FYN**	**1.72**	**RIPK2**	**2.04**		
		**RIPK2**	**1.68**	**PLAU**	**2.02**		
		**RICTOR**	**1.59**	**TGFBR2**	**1.97**		
		**SMAD2**	**1.56**	**MET**	**1.86**		
		**MAPK8**	**1.55**	**TLR2**	**1.85**		
		**CREB1**	**1.53**	**IRF1**	**1.84**		
		MIR31	-1.55	**HS3ST1**	**1.83**		
		DACH1	-1.56	**CSF1**	**1.83**		
		Trib2	-1.59	**TGFA**	**1.73**		
		HRAS	-1.61	**ESM1**	**1.72**		

	JAG2	-1.63	**BIRC3**	**1.62**	
	MCM6	-1.63	**IL6**	**1.61**	
	CNTN1	-1.84	**CSF2**	**1.57**	
	PTHLH	-1.87	**SPP1**	**1.56**	
	DNMT1	-1.93	**BMI1**	**1.53**	
	KAT2A	-1.95	**BMP6**	**1.51**	
	TFRC	-2.02	STAT6	-1.53	
	MKI67	-2.33	ID3	-1.54	
	LRP8	-2.40	MIR31	-1.56	
	IGFBP5	-3.40	LRP8	-1.63	
			HRAS	-1.77	
			LRP1	-1.80	
			JAG2	-1.83	
			PTHLH	-1.96	
			NOTCH1	-2.11	
			HEY1	-2.32	
			IGFBP5	-2.55	

Supplemental Table 4. Aldehyde dehydrogenase signaling. Differentially expressed (p≤0.01 and 1.5-fold) at a minimum of one time point in UT14 xenografts after radiation. Bold is upregulated after XRT; red, downregulated.

Day 4 vs Controls		Day 7 vs Controls		Day 12 vs Controls		Day 21 vs Controls	
**XDH**	**2.06**	**ALDH1A3**	**2.18**	**IL8**	**6.00**	**ALDH1A3**	**2.75**
**CTNNB1**	**1.63**	**SPG21**	**1.64**	**ALDH1A3**	**4.18**	NOTCH1	-1.69
**SPG21**	**1.57**	PSIP1	-1.70	**EPCAM**	**2.10**		
		ALDH1B1	-2.09	**CSF2**	**1.57**		
		CBR1	-2.22	NOTCH1	-2.11		

Supplemental Table 5. BMI1 signaling. Differentially expressed (p≤0.01 and 1.5-fold) at a minimum of one time point in UT14 xenografts after radiation. Bold is upregulated after XRT; red, downregulated.

**Day 4 vs Controls**		**Day 7 vs Controls**		**Day 12 vs Controls**		**Day 21 vs Controls**	
**CTNNB1**	**1.63**	**WNT7A**	**2.46**	**MMP9**	**2.82**	NOTCH1	-1.69
		**SMAD2**	**1.56**	**WNT7A**	**2.25**		
		MEN1	-1.54	**PMAIP1**	**2.04**		
		DNMT1	-1.93	**IL6**	**1.61**		
		UHRF1	-2.12	**SPP1**	**1.56**		
		PLK1	-2.20	**BMI1**	**1.53**		
		MKI67	-2.33	NOTCH1	-2.11		
		PRC1	-2.37				
		EGR1	-2.55				

Supplemental Table 6. CD133 (PROM1) signaling. Differentially expressed (p≤0.01 and 1.5-fold) at a minimum of one time point in UT14 xenografts after radiation. Bold is upregulated after XRT; red, downregulated.

Day 4 vs Controls		Day 7 vs Controls		Day 12 vs Controls		Day 21 vs Controls	
**CTNNB1**	**1.63**	**TIMP2**	**2.39**	**IL8**	**6.00**	IL6R	-1.55
**MMP28**	**1.52**	**CTSB**	**1.83**	**CXCL1**	**3.16**	NOTCH1	-1.69
FASN	-2.06	**MAPK8**	**1.55**	**TIMP2**	**2.92**	FASN	-1.74
		SLC9A3R1	-1.79	**MMP9**	**2.82**		
		CNTN1	-1.84	**HBEGF**	**2.76**		
		TFRC	-2.02	**EPCAM**	**2.10**		
		FASN	-2.54	**IL6**	**1.61**		
				**CSF2**	**1.57**		
				**BMI1**	**1.53**		
				LRP1	-1.80		
				FASN	-1.88		
				NOTCH1	-2.11		

Supplemental Table 7. NANOG signaling. Differentially expressed (p≤0.01 and 1.5-fold) at a minimum of one time point in UT14 xenografts after radiation. Bold is upregulated after XRT; red, downregulated.

Day 4 vs Controls		Day 7 vs Controls		Day 12 vs Controls		Day 21 vs Controls	
**TOR1AIP2**	**3.50**	**TOR1AIP2**	**4.27**	**IL8**	**6.00**	**LYN**	**2.19**
**ICAM1**	**2.58**	**PMP22**	**3.32**	**TOR1AIP2**	**3.75**	**PMP22**	**1.95**
**LYN**	**2.02**	**NFATC2**	**2.04**	**ICAM1**	**3.51**	**TFCP2L1**	**1.94**
**CTNNB1**	**1.63**	**TGFA**	**1.91**	**LIF**	**3.13**	**CDK6**	**1.54**
**CDK6**	**1.61**	**SEMA6A**	**1.90**	**LYN**	**3.07**	KLF6	-1.54
DACH1	-1.59	**MET**	**1.76**	**PMP22**	**3.02**	ARID3A	-1.57
IGFBP5	-1.85	**SMAD2**	**1.56**	**MMP9**	**2.82**	NOTCH1	-1.69
		DACH1	-1.56	**IL6ST**	**2.11**		
		JAG2	-1.63	**EPCAM**	**2.10**		
		DNMT1	-1.93	**TGFBR2**	**1.97**		
		IGFBP5	-3.40	**MET**	**1.86**		
				**TGFA**	**1.73**		
				**IL6**	**1.61**		
				**BMI1**	**1.53**		
				**BMP6**	**1.51**		
				JAG2	-1.83		
				LDB1	-2.00		
				NOTCH1	-2.11		
				IGFBP5	-2.55		

Supplemental Table 8. POU5F1 (Oct3/4) signaling. Genes differentially expressed (p≤0.01 and 1.5-fold) at a minimum of one time point in UT14 xenografts after radiation. Bold is upregulated after XRT; red, downregulated.

**Day 4 vs Controls**		**Day 7 vs Controls**		**Day 12 vs Controls**		**Day 21 vs Controls**	
**TOR1AIP2**	**3.50**	**TOR1AIP2**	**4.27**	**IL8**	**6.00**	IGFBP7	2.21
**ICAM1**	**2.58**	**NFATC2**	**2.04**	**TOR1AIP2**	**3.75**	TFCP2L1	1.94
**CTNNB1**	**1.63**	**IGFBP7**	**1.96**	**ICAM1**	**3.51**	IL32	1.89
**CDK6**	**1.61**	**TGFA**	**1.91**	**LIF**	**3.13**	CDK6	1.54
**KPNA1**	**1.55**	**SEMA6A**	**1.90**	**MMP9**	**2.82**	ARID3A	-1.57
**PPIA**	**1.54**	**GADD45A**	**1.85**	**HBEGF**	**2.76**	IRS1	-1.66
DACH1	-1.59	**MET**	**1.76**	**IL32**	**2.67**	NOTCH1	-1.69
		**JMJD1C**	**1.63**	**IGFBP7**	**2.37**	AHCY	-1.71
		**SMAD2**	**1.56**	**IL6ST**	**2.11**		
		**MAPK8**	**1.55**	**EPCAM**	**2.10**		
		DACH1	-1.56	**TGFBR2**	**1.97**		
		JAG2	-1.63	**MET**	**1.86**		
		IRS1	-1.64	**TGFA**	**1.73**		
		DNMT1	-1.93	**GADD45A**	**1.62**		
		MKI67	-2.33	**IL6**	**1.61**		
				**CSF2**	**1.57**		
				**SPP1**	**1.56**		
				**BMI1**	**1.53**		
				JAG2	-1.83		
				IRS1	-1.84		
				LDB1	-2.00		
				NOTCH1	-2.11		

Supplemental Table 9. SOX2 signaling. Genes differentially expressed (p≤0.01 and 1.5-fold) at a minimum of one time point in UT14 xenografts after radiation. Bold is upregulated after XRT; red, downregulated.

**Day 4 vs Controls**		**Day 7 vs Controls**		**Day 12 vs Controls**		**Day 21 vs Controls**	
**ICAM1**	**2.58**	**MMP3**	**1.99**	**IL8**	**6.00**	**TFCP2L1**	**1.94**
**CTNNB1**	**1.63**	**TGFA**	**1.91**	**ICAM1**	**3.51**	ARID3A	-1.57
**KPNA1**	**1.55**	**SEMA6A**	**1.90**	**LIF**	**3.13**	NOTCH1	-1.69
DACH1	-1.59	**SMURF1**	**1.80**	**MMP9**	**2.82**		
		**HDAC9**	**1.79**	**HDAC9**	**2.19**		
		**MET**	**1.76**	**EPCAM**	**2.10**		
		**SMAD2**	**1.56**	**TGFBR2**	**1.97**		
		**MAPK8**	**1.55**	**MET**	**1.86**		
		SRRT	-1.51	**TGFA**	**1.73**		
		DACH1	-1.56	**SPP1**	**1.56**		
		PLK1	-2.20	**BMI1**	**1.53**		
		MKI67	-2.33	**BMP6**	**1.51**		
		PTTG1	-3.08	STAT6	-1.53		
				NOTCH1	-2.11		
				HEY1	-2.32		

Supplemental Table 10. Epithelial to mesenchymal transition (EMT) related signaling. Genes differentially expressed (p≤0.01 and 1.5-fold) at a minimum of one time point in UT14 xenografts after radiation. Bold is upregulated after XRT; red, downregulated.

Day 4 vs Controls		Day 7 vs Controls		Day 12 vs Controls		Day 21 vs Controls	
**C3**	**6.77**	**TIMP3**	**2.44**	**LCN2**	**10.0**	**C3**	**5.02**
**ICAM1**	**2.58**	**TIMP2**	**2.39**	**C3**	**6.38**	**HAS2**	**4.82**
**XDH**	**2.06**	**DAB2**	**2.12**	**IL8**	**6.00**	**DAB2**	**2.29**
**LYN**	**2.02**	**NFATC2**	**2.04**	**HAS2**	**5.35**	**LYN**	**2.19**
**SOD2**	**1.79**	**MMP3**	**1.99**	**CEACAM6**	**4.80**	**CDH11**	**1.85**
**DAB2**	**1.77**	**TGFA**	**1.91**	**DAB2**	**4.24**	**CDK6**	**1.54**
**CTNNB1**	**1.63**	**PTGES**	**1.86**	**ICAM1**	**3.51**	KLF6	-1.54
**CDK6**	**1.61**	**GADD45A**	**1.85**	**PLAUR**	**3.32**	IL6R	-1.55
**RICTOR**	**1.56**	**PLAUR**	**1.84**	**LIF**	**3.13**	MTSS1	-1.57
**CREB1**	**1.55**	**CDH13**	**1.80**	**LYN**	**3.07**	PVRL1	-1.59
**CCNG1**	**1.55**	**SMURF1**	**1.80**	**TIMP2**	**2.92**	ZBTB7A	-1.66
ZFPM2	-1.59	**BCAR3**	**1.80**	**MMP9**	**2.82**	IRS1	-1.66
IGFBP5	-1.85	**MET**	**1.76**	**HBEGF**	**2.76**	NOTCH1	-1.69
FASN	-2.06	**FYN**	**1.72**	**CXCL16**	**2.64**	HRAS	-1.71
PTHLH	-2.17	**TRAF6**	**1.70**	**MME**	**2.17**	FASN	-1.74
		**ANGPTL4**	**1.69**	**TRIB3**	**2.10**	TNS4	-1.76
		**VCAN**	**1.69**	**EPCAM**	**2.10**		
		**RICTOR**	**1.59**	**PMEPA1**	**2.05**		
		**GALNT3**	**1.56**	**PLAU**	**2.02**		
		**SMAD2**	**1.56**	**SEMA7A**	**1.99**		
		**NRAS**	**1.55**	**TGFBR2**	**1.97**		
		**MAPK8**	**1.55**	**FAP**	**1.91**		
		**CREB1**	**1.53**	**SOD2**	**1.87**		

	**ADRB2**	**1.53**	**MET**	**1.86**	
	MIR31	-1.55	**CEACAM1**	**1.85**	
	HRAS	-1.61	**CDH11**	**1.83**	
	JAG2	-1.63	**GDF15**	**1.82**	
	IRS1	-1.64	**TGFA**	**1.73**	
	SLC9A3R1	-1.79	**ESM1**	**1.72**	
	PTHLH	-1.87	**STC2**	**1.71**	
	DNMT1	-1.93	**BCAR3**	**1.66**	
	UHRF1	-2.12	**GADD45A**	**1.62**	
	CBR1	-2.22	**IL6**	**1.61**	
	FASN	-2.54	**TRAF6**	**1.58**	
	EGR1	-2.55	**CSF2**	**1.57**	
	PTTG1	-3.08	**SPP1**	**1.56**	
	IGFBP5	-3.40	**BMI1**	**1.53**	
			**BMP6**	**1.51**	
			ID3	-1.54	
			MIR31	-1.56	
			MTSS1	-1.58	
			IDH2	-1.58	
			SEMA4C	-1.61	
			PVRL1	-1.66	
			MAP3K4	-1.68	
			SDC1	-1.70	
			HRAS	-1.77	
			JAG2	-1.83	
			IRS1	-1.84	
			FASN	-1.88	
			PTHLH	-1.96	

		CSPG4 -2.09<br>NOTCH1 -2.11<br>PTPRZ1 -2.12<br>HEY1 -2.32<br>IGFBP5 -2.55	
